# The Use and Abuse of Medical Literature

**Published:** 1921-07-23

**Authors:** 


### THE USE AND ABUSE OF MEDICAL LITERATURE.

The medical world is indebted to Professor Bullock for a delightful lecture, recently delivered at St. Mary's Hospital. It is good for all of us at times to be subjected to good-natured criticism, and on this occasion there were few corners in either the editors' or the contributors' sphere which did not justifiably receive a delicate thrust. In the young house surgeon who proposed to write a paper on the injuries of the upper extremity, but received the sound advice that he should confine himself to the last phalanx of the little finger and find sufficient material, one sees a humorously typical instance of an error into which so many of us may fall. How often is a writer careful to review with even approximate perfection the standing literature of the subject upon which he threatens to discourse? Yet Professor Bullock gave some almost startling figures to prove the remarkable output of the medical pen. Would that as a profession we could claim it all as representing original thought. Between 1893 and 1913, according to the *Catalogue of the Surgeon-General's Library* at Washington—the greatest medical library in the world—25,000 articles were written on tuberculosis alone—half of them on the pulmonary form! Up to 1893 some 7,000 articles and books were produced on syphilis, yet in the next twenty years this number was doubled. In the same twenty years 7,000 contributions were devoted to heart disease, and 7,000 to pregnancy. Well might the lecturer ask what the result would be if this colossal production continued in the same ratio of increase. At no distant date our libraries might be great cities, where those who did not write must edit and catalogue!

Professor Bullock was not kind to the weekly journals. In a satirical summary their contents may not sound attractive, but the records must be kept. A voluminous lecture by a "leader of the profession" is not necessarily good. Sometimes it may be truly bad, if judged by the ultra-modern, but at the same time reviews and repetitions have their value where an established truth has to be driven home. We would heartily agree, however, with the lecturer's plea for brevity. In the world of medical literature no other virtue seems so readily lost.

Nevertheless, as Professor Bullock explained, medical periodical literature is a necessity. Medical men should not, indeed cannot, be individualists. They must supplement their own work with the experience of others. The result in a busy world is perhaps inevitable, and notwithstanding the great literature existing, there are vast lacunæ in our knowledge whichever way we turn. To close them or, more strictly, to prevent their increase, we must perfect that literature in many small but vital ways. Accuracy of reference and conciseness of titles are both obvious necessities. It is undoubtedly a fact that time and again affirmations are made in medical literature which have never been truly tested and would not indeed withstand the process. Among a host of other points, the speaker urged that all articles should be signed. He feels that anonymous articles, or articles with the initials only appended, suggest that the author has something to conceal! He desires to see yet more editorial supervision and censorship, and, above all, he deplores the publication of the same paper in several journals. In the library of the Royal College of Surgeons he had spread out before him not long ago six separate journals all containing the same paper. He checked them paragraph by paragraph. Indeed, there are lacunæ in our medical literature and in all its phases, but we know its latest critic as its staunch supporter too.

